# PspC domain-containing protein (PCP) determines *Streptococcus mutans* biofilm formation through bacterial extracellular DNA release and platelet adhesion in experimental endocarditis

**DOI:** 10.1371/journal.ppat.1009289

**Published:** 2021-02-12

**Authors:** Chiau-Jing Jung, Chih-Chieh Hsu, Jeng-Wei Chen, Hung-Wei Cheng, Chang-Tsu Yuan, Yu-Min Kuo, Ron-Bin Hsu, Jean-San Chia

**Affiliations:** 1 Department of Microbiology and Immunology, School of Medicine, College of Medicine, Taipei Medical University, Taipei, Taiwan; 2 Graduate Institute of Oral Biology, School of Dentistry, National Taiwan University, Taipei, Taiwan; 3 Division of Cardiovascular Surgery, Department of Surgery, National Taiwan University Hospital, Taipei, Taiwan; 4 Graduate Institute of Clinical Medicine, College of Medicine, National Taiwan University, Taipei, Taiwan; 5 Graduate Institute of Microbiology, College of Medicine, National Taiwan University, Taipei, Taiwan; 6 Department of Pathology, National Taiwan University Cancer Center, Taipei, Taiwan; 7 Department of Pathology, National Taiwan University Hospital, Taipei, Taiwan; 8 Graduate Institute of Immunology, College of Medicine, National Taiwan University, Taipei, Taiwan; The University of Alabama at Birmingham, UNITED STATES

## Abstract

Bacterial extracellular DNA (eDNA) and activated platelets have been found to contribute to biofilm formation by *Streptococcus mutans* on injured heart valves to induce infective endocarditis (IE), yet the bacterial component directly responsible for biofilm formation or platelet adhesion remains unclear. Using *in vivo* survival assays coupled with microarray analysis, the present study identified a LiaR-regulated PspC domain-containing protein (PCP) in *S*. *mutans* that mediates bacterial biofilm formation *in vivo*. Reverse transcriptase- and chromatin immunoprecipitation-polymerase chain reaction assays confirmed the regulation of *pcp* by LiaR, while PCP is well-preserved among streptococcal pathogens. Deficiency of *pcp* reduced *in vitro* and *in vivo* biofilm formation and released the eDNA inside bacteria floe along with reduced bacterial platelet adhesion capacity in a fibrinogen-dependent manner. Therefore, LiaR-regulated PCP alone could determine release of bacterial eDNA and binding to platelets, thus contributing to biofilm formation in *S*. *mutans*-induced IE.

## Author summary

Phage shock proteins (Psps) are well-characterized in Gram-negative bacteria, yet their roles in Gram-positive were yet unknown. Interestingly, we found a PspC homolog [named PspC domain-containing protein (PCP) in this study] that is well preserved and conserved among pathogenic streptococci, including *S*. *pneumonia* and group A and group B streptococci. Using a rat experimental endocarditis infection model and *S*. *mutans* as a representative pathogen, we demonstrated PCP alone could mediate bacterial colonization and biofilm formation *in situ*. Therefore, PCP codes for an essential gene controlling biofilm formation *in vivo* for *S*. *mutans* and possibly other pathogenic streptococci.

## Introduction

Bacterial biofilm formation determines not only pathogenesis, but also antibiotic resistance upon colonizing host microenvironments to cause infectious diseases. Yet, the adaptation of bacteria or components responsible for forming biofilm *in vivo* have remained largely unclear. A typical example is infective endocarditis (IE), which is a biofilm-associated disease and most frequently caused by staphylococci or oral streptococci [1,2]. Previously, we demonstrated these IE pathogens can hijack platelets and neutrophil extracellular traps (NETs) to promote biofilm and vegetation formation [3–5]. In addition, plasma components can enhance *Streptococcus mutans* survival in the blood circulation and modulate bacterial biofilm formation through enhancing autolysin maturation for releasing bacterial extracellular DNA (eDNA), which is an important matrix component of biofilm on the damaged heart valve *in vivo* [6]. These data suggested that host factors can modulate bacterial virulence causing IE, but the bacterial regulatory components responsible for regulating bacterial virulence for causing IE remain unclear.

Bacteria adapt to environmental stimulations and regulate downstream gene expression through two component systems (TCSs) [7]. TCSs typically consist of a sensor kinase and a response regulator, which is generally a DNA-binding protein that modulates the expression of target genes [8,9]. Although sensor kinases and response regulators are usually paired and localized in operons, they also crosstalk with other TCSs for regulating bacterial gene expression in response to a variety of environmental stimuli. Bioinformatic analysis of the *S*. *mutans* UA159 genome sequence identified 13 putative TCS (TCS-1 to TCS-13) and one orphan response regulator (GcrR or CovR) in *S*. *mutans* [10]. Among these TCSs, the role of several in bacterial biofilm formation has been demonstrated, including TCS-1 (VicKR) [11], TCS-2 (CiaHR) [10], TCS11 (LiaRS) [12], TCS-13 (ComDE) [13], and GcrR [14]. *In vitro*, these TCSs have been shown to regulate biofilm formation by regulating glucosyltransferase (Gtf) systems, which control the sucrose-mediated glucan production crucial for *S*. *mutans* biofilm formation on polystyrene and dental surfaces [11,15]. However, there is no sucrose in plasma, and glucosyltransferase-deficient strains do not reduce their abilities to cause IE [16], suggesting *S*. *mutans* exploit other bacterial or host components, instead of glucan, to form biofilms on heart valves in IE. Consistently, our previous study demonstrated that platelets and bacterial eDNA play important roles in *S*. *mutans* biofilm formation on the heart valve in IE [6]. The data suggested novel TCS-regulated components in *S*. *mutans* may determine biofilm formation *in vivo*.

In this study, using *in vivo* survival assay and microarray analysis, we identified LiaR plays an essential role in biofilm formation on damaged heart valves *in situ* in an experimental IE rat model. The underlying mechanisms mainly involved novel phage shock protein C (PspC) domain-containing protein (PCP)-mediated eDNA release and platelet adhesion capacities. In addition, PCP is well conserved in other streptococcal pathogens, implying their essential roles in pathogenesis through modulating biofilm formation.

## Results

### *LiaR*-deficient S. *mutans* has reduced ability to cause vegetation *in vivo*

**Fig 1:**
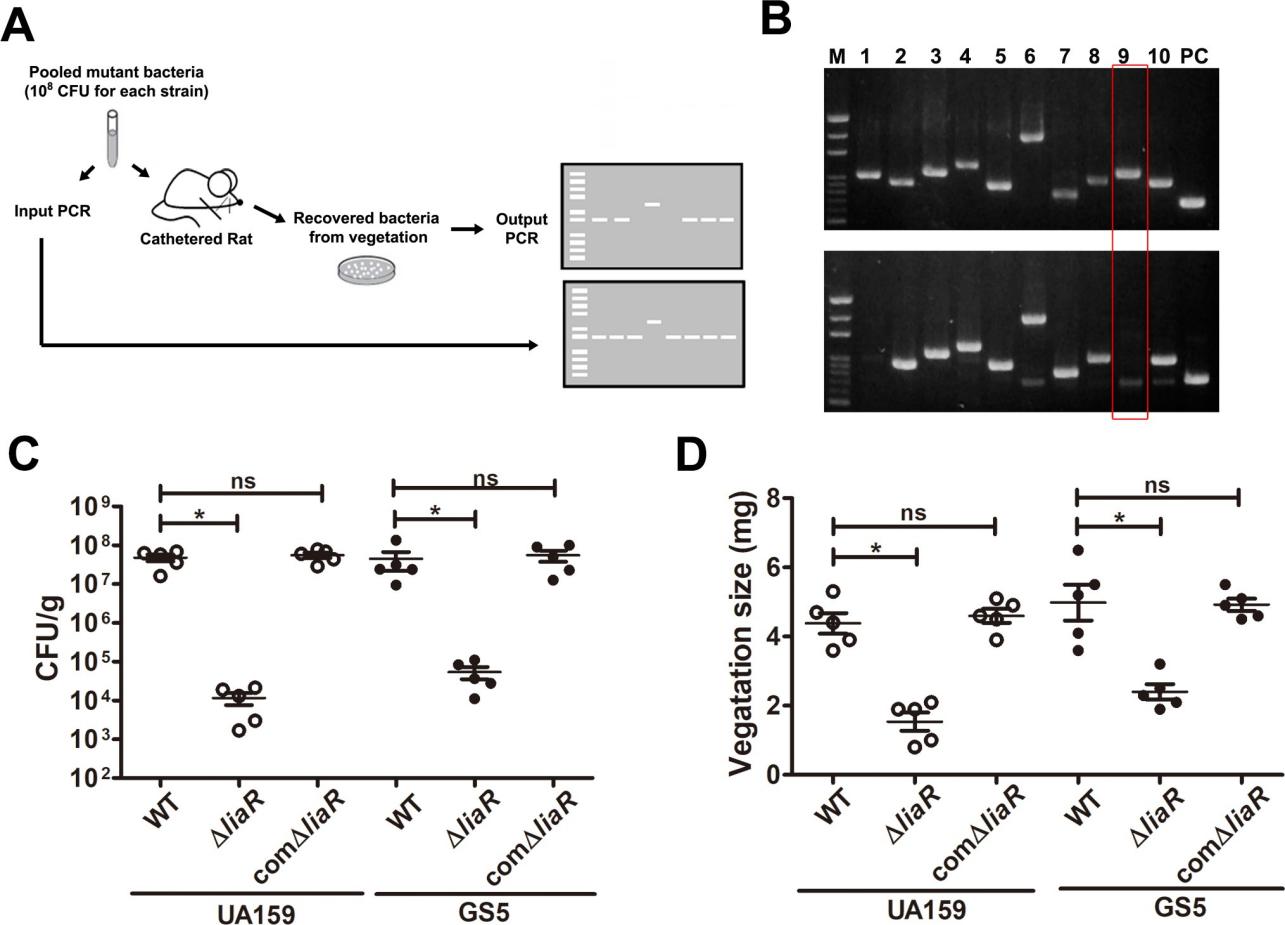
*LiaR*-deficient mutant strain shows reduce ability to cause vegetation formation in experimental rat IE model. (A) Schematic diagram of *in vivo* survival assay. A mix of 10 insertion mutants of transcription regulators was injected (10^8^ for each) into catharized rats, and the bacteria colonized inside the vegetation on damaged valves were identified by specific primers. (B) The upper panel is a representative PCR result of injected bacterial mixtures (input PCR); the bottom panel is a representative PCR result of bacteria colonized inside the vegetation. M, DNA marker; lane 1, the PCR result using the primers for detecting *SMU*.*1146*-deficient mutant; lane 2, *SMU*.*927*; lane 3, *SMU*.*1815*; lane 4, *SMU*.*659*; lane 5, *SMU*.*1038*; lane 6, *SMU*.*1008*; lane 7, *SMU*.*1964*; lane 8, *SMU*.*576*; lane 9, *SMU*.*487* (LiaR); lane 10, *SMU*.*1547*; and PC, positive control, PCR product of 16S rRNA gene. (C and D) The role of LiaR in the pathogenesis of *S*. *mutans*-induced IE was investigated using Δ*liaR* and comΔ*liaR* strains of GS5 and UA159 in experimental rat endocarditis models. The number of colonized bacteria inside vegetations (C) and vegetation size (D) were measured. Data represent the means ± standard error of the means and were statistically analyzed using the Kruskal-Wallis test with subsequent Dunn’s test; *P < 0.05, ns, not significant. IE, infective endocarditis; PCR, polymerase chain reaction; and CLSM, confocal laser scanning microscopy.

Two component systems sense environmental cues and responses by rapidly regulating gene expression. To identify essential genes of *S*. *mutans in vivo*, an *in situ* biofilm formation was screened on damaged heart valves using a panel of isogenic mutant strains of *S*. *mutans* TCS with unknown functions, including SMU.1146, SMU.927, SMU.1815, SMU.659, SMU.1038, SMU.1008, SMU.1964, SMU.576, SMU.487, and SMU.1547 [10] via intravenous infection (Fig 1A). Briefly, a mixture of 10 transcription regulator isogenic mutant strains of *S*. *mutans* [10^8^ colony forming unit (CFU)/each] were infected into catheterized rats, and each mutant strain in the mixture could be recognized by specific primers (Table 1; Fig 1B, upper panel). Twenty-four hours after infection, the bacteria colonized inside the vegetation were harvested and identified using specific primers (Fig 1B, bottom panel). Interestingly, SMU.487 (also named *liaR*)-deficient bacteria were hardly found in the harvested bacterial mixture. To confirm the role of LiaR in the pathogenesis of *S*. *mutans*-induced IE, the *liaR*-deficient strains of *S*. *mutans* UA159 and GS5 were tested for their virulence in the experimental rat IE model. *LiaR*-deficient strains exhibited a significantly reduced ability to colonize on heart valve and form biofilm-related septic thrombi (vegetation) compared with the parental strain (Fig 1C and 1D, S1 Fig and S2 Fig). Complementation of the *liaR* gene into *liaR*-deficient strains restored the phenotype, confirming the role of LiaR (Fig 1C and 1D, S1 Fig and S2 Fig). These data suggest LiaR regulates *S*. *mutans* virulence in the pathogenesis of IE.

**Table 1:** PCR primers used in the current study.

Primers	Sequence (5’ to 3’) (restriction sites: underlined)	Target
**For bacteria identification in *in vivo* survival assay**		
EMR	GCGTGTTCATTGCTTGA	Erythromycin cassette
EMF	CCGTTTACGAAATTGGAAC	
SMU.1146-R	AAATCGTTTAGTGTCCTGTC	with EMF; SMU.1146-insetion mutant
SMU.927-F	CGATATCAAGAACCCAGA	with EMR; SMU.927-insertion mutant
SMU.1815-R	GTCCTAGCTAAACGTGGA	with EMF; SMU.1815-insertion mutant
SMU.659-F	CATTATGGATGGTGGAAC	with EMR; SMU.659-insertion mutant
SMU.1038-R	GCTTGCTGGCTATAGATG	with EMF; SMU.1038-insertion mutant
SMU.1008-F	CTTTCAGAAGGAACACAAG	with EMR; SMU.1008-insertion mutant
SMU.1964-F	AATAAGGTTAACAGGGCTC	with EMR; SMU.1964-insertion mutant
SMU.576-F	ATAAGCTGAGAAACGAGC	with EMR; SMU.576-insertion mutant
SMU.487-R	AGCTTTGTCTTTACCAGC	with EMF; SMU.487-insertion mutant
SMU.1547-F	CCGTCTATCAACCTTAGC	with EMR; SMU.1547-insertion mutant
SMU.1128-F	GCTTCTTCAAGTATCGACA	with EMR; SMU.1128-insertion mutant
SMU.1145-F	TTACGAAGCTTGAAGACTG	with EMR; SMU.1145-insertion mutant
SMU.1814-F	ATGCCAATGACTTACTGC	with EMR; SMU.1814-insertion mutant
SMU.1037-F	CTCGGATTTATCGTCATC	with EMR; SMU.1037-insertion mutant
SMU.1009-R	GTAAGTGAACGGAACAGAA	with EMF; SMU.1009-insertion mutant
SMU.1965-R	GTTGTGCCCGTTGAATTAA	with EMF; SMU.1965-insertion mutant
SMU.577-R	CAGCAGTTCATTTATCTCC	with EMF; SMU.577-insertion mutant
SMU.486-F	ATCTATGGCTATGTTCGC	with EMR; SMU.486-insertion mutant
SMU.1548-R	GACAGCTACCGGTCTTAC	with EMF; SMU.1548-insertion mutant
**For generation of deletion mutants and the complementation strains**		
*liaR*-BF	GCTCAGAATGACTTGCGAG	upstream fragment of *liaR*
*liaR*-BR-EcoRI	GGAATTCCTTTTGTTTTACTCATCAGCA	
*liaR*-AF-EcoRI	GGAATTCCATCATTTAGTGCCACAGG	downstream fragment of *liaR*
*liaR*-AR	AGCTTTGTCTTTACCAGC	
*pcp*-BF	ACAAACAACAGGCGGAC	upstream fragment of *pcp*
*pcp*-BR-*EcoR*I	GGAATTCCACAACTCCGCAGATCA	
*pcp*-AF-*EcoR*I	GGAATTCCATCGTCATGCTCAAGGG	downstream fragment of *pcp*
*pcp*-AR	GATAATGGCTGGTGTTTCA	
*pcp*PF-*Xba*I	GCTCTAGAGCTGAATCGTGTGGTCAAGT	*pcp*
*pcp*R-*Xba*I	GCTCTAGAGCTTACCAAAACCATTTGTTAT	
**Construction of recombinant LiaR**		
*liaR*F-BamHI	CGGGATCCCGTGCTGATGAGTAAAACAAAA	*liaR*
*liaR*R-BamHI	CGGGATCCCGCTAATTTTCGTCCTGTGG	
**RT-PCR and CHIP-PCR of *pcp***		
*pcp*F	GGTAGTATGATCTGCGGA	*pcp*
*pcp*R	CCTTGAGCATGACGATAA	
16S-F	AGAGTTTGATCMTGGCTCAG	16S rRNA gene
16S-R	GGTTACCTTGTTACGACTT	

### LiaR regulates PCP-mediated biofilm formation *in vivo*

**Fig 2:**
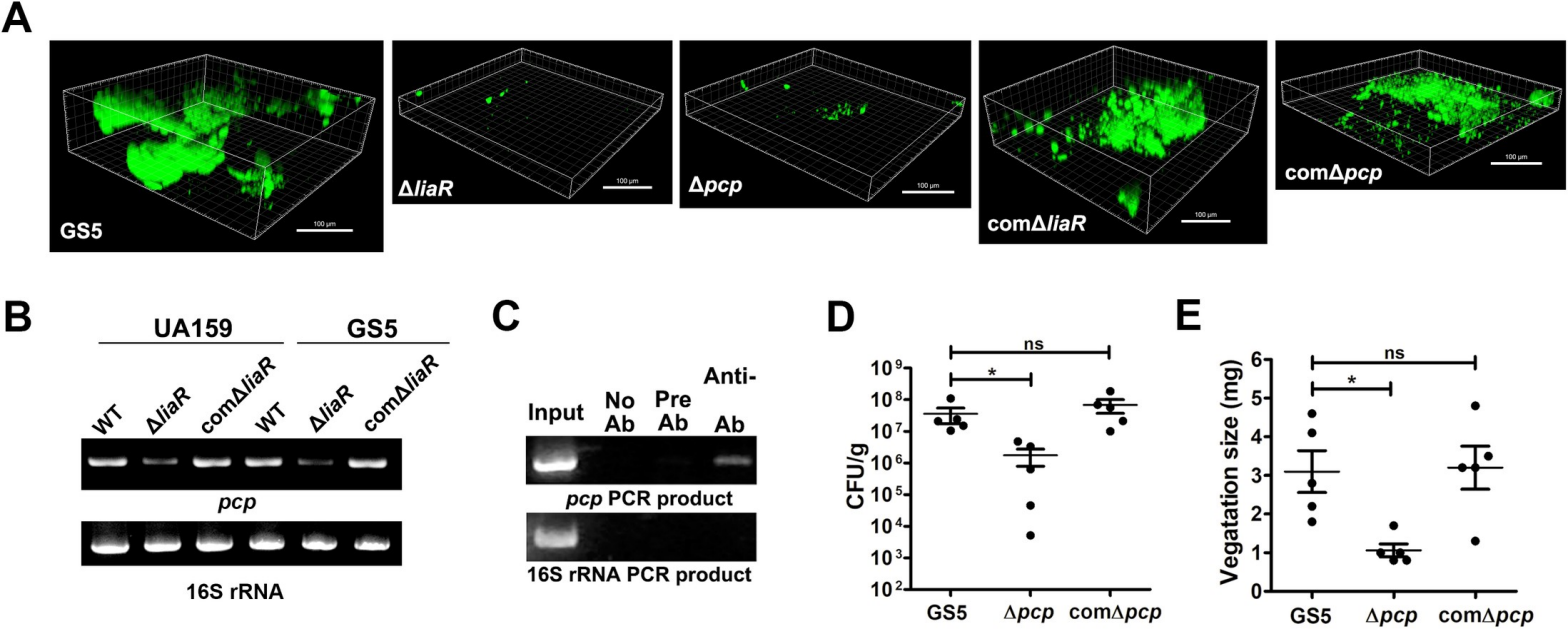
LiaR regulates expression of PCP, which contributes to *S*. *mutans* biofilm formation on damaged valves *in vivo*. **. (A)** GFP-tagged *S*. *mutans* GS5 wild type, Δ*liaR*, Δ*pcp*, comΔ*liaR*, or comΔ*pcp* strains were intravenously injected into experimental IE rat models, and the biofilm inside vegetations harvested from injured heart valves were observed by CLSM (630× magnification). The data represent the three-dimensional structure of the *S*. *mutans* biofilm inside the rat heart valve vegetation *in vivo*. These experiments were repeated at least three times, with a representative experiment shown. (B) Expression of *pcp* in *S*. *mutans* wild type, Δ*liaR* and comΔ*liaR* strains were measured by RT-PCR. The experiment was repeated three times, with a representative experiment shown. (C) The binding of LiaR on the *pcp* region was detected by ChIP assay. The PCR product of the 16S rRNA gene was used as a negative control. The experiment was repeated three times, with a representative experiment shown. (D and E) The role of PCP in the pathogenesis of *S*. *mutans*-induced IE was investigated using Δ*pcp* and comΔ*pcp* mutant strains in the experimental rat IE model. The number of colonized bacteria inside vegetations (D) and vegetation size (E) were measured. Data represent the means ± standard error of the means and were statistically analyzed using the Kruskal-Wallis test with subsequent Dunn’s test; *P < 0.05; ns, not significant. IE, infective endocarditis; GFP, green fluorescent protein; CLSM, confocal laser scanning microscopy; RT-PCR, reverse transcription-polymerase chain reaction; ChIP, chromatin immunoprecipitation; and PCR, polymerase chain reaction.

**Fig 3:**
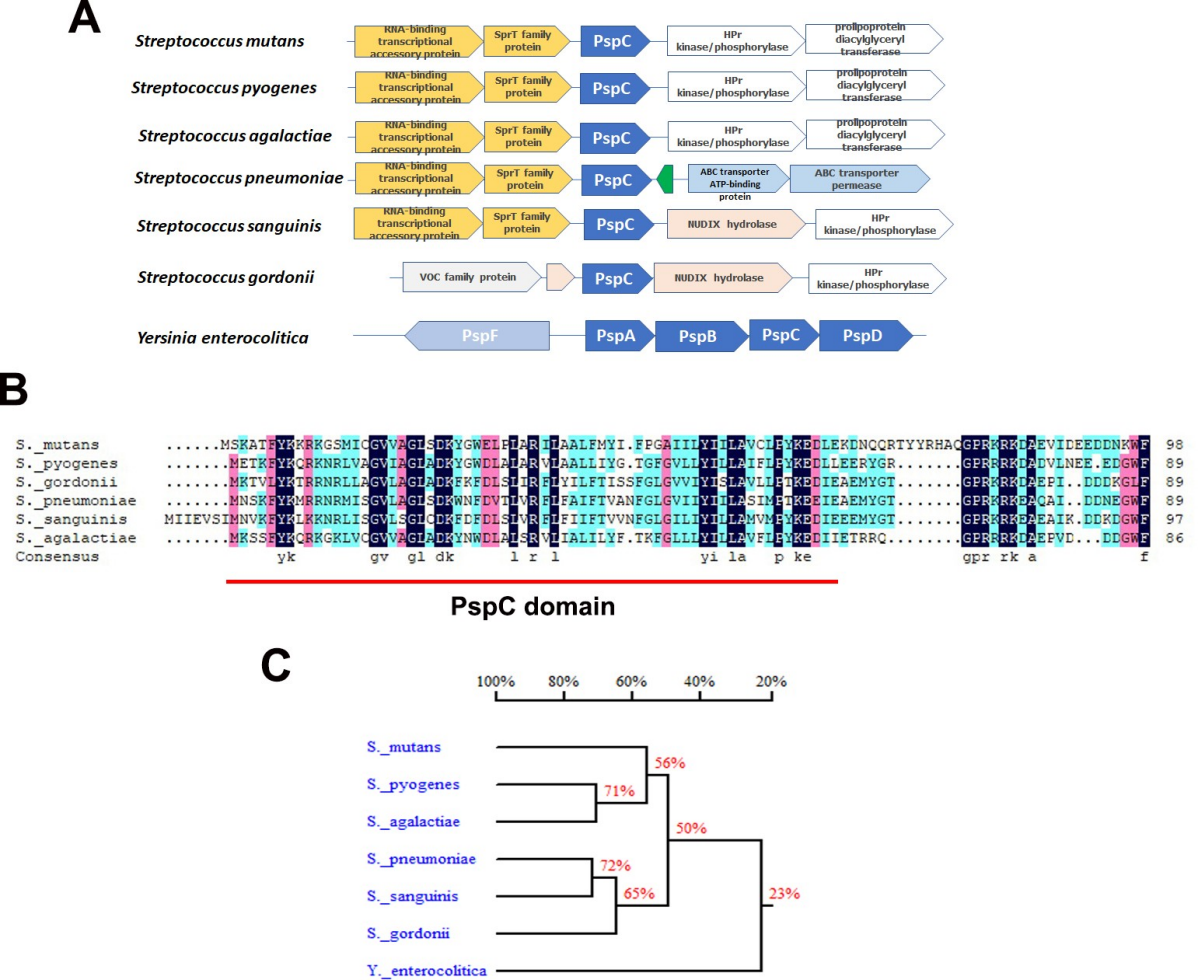
Good preservation of PCP among pathogenic streptococci. (A) The schematic illustrates upstream and downstream open reading frames and orientations of PCP among pathogenic streptococci. None of the other Psp stress operon genes were observed. (B) Alignment of the amino acid sequence of PCP among pathogenic streptococci, obtained from the NCBI database. Good preservation of the amino acid sequence was observed. (C) Homology tree of the amino acid sequences of the PCP among pathogenic streptococci.

To observe bacterial biofilm formation on damaged heart valves *in vivo*, a green fluorescent protein (GFP)-tagged *S*. *mutans* GS5 strain was used in the experimental IE model [3]. Consistently, the *liaR*-deficient strain showed reduced ability to form biofilms on the damaged valve *in situ* compared with the parental strain (Fig 2A and S3 Fig). To identify LiaR-regulated downstream genes, microarray analysis was performed (S1 Table). Notably, among the LiaR-regulated genes, expression of *SMU*.*753*, which encodes a PspC domain-containing protein (named PCP here) [17], was dramatically reduced in the *liaR*-deficient mutant strain (S1 Table). Data from reverse transcriptase (RT)- and chromatin immunoprecipitation (ChIP)-polymerase chain reaction (PCR) assays confirmed LiaR can directly bind the promoter region to regulate expression of *pcp* (Fig 2B and 2C). The *Pcp*-deficient mutant also showed a reduced ability to colonize and form a biofilm on the damaged valve (Fig 2A, 2D and S3 Fig), with reduced vegetation formation size (Fig 2E, S1 Fig and S2 Fig) in the experimental rat IE model. PspC is a member of the Psp stress response system, which contains four core components (PspF, -A, -B, and -C) and modulates the bacterial stress response in Gram-negative bacteria, such as *Escherichia coli* and *Yersinia enterocolitica* [18]. However, *S*. *mutans* and other streptococci only contained a PspC homolog (PCP) and did not have other Psp operon genes compared with *Y*. *enterocolitica* (Fig 3A). A comparison of amino acid sequences with PCPs of the other pathogen streptococci showed good-preservation among these pathogenic streptococci (Fig 3B and 3C). These data suggested that the LiaR-regulated protein, PCP, mediates *in vivo S*. *mutans* biofilm and vegetation formation, and preservation of PCP implied this same role in biofilm formation of other streptococci.

### PCP modulates bacterial eDNA release, which contributes to bacterial biofilm formation *in vitro* and *in vivo*

**Fig 4:**
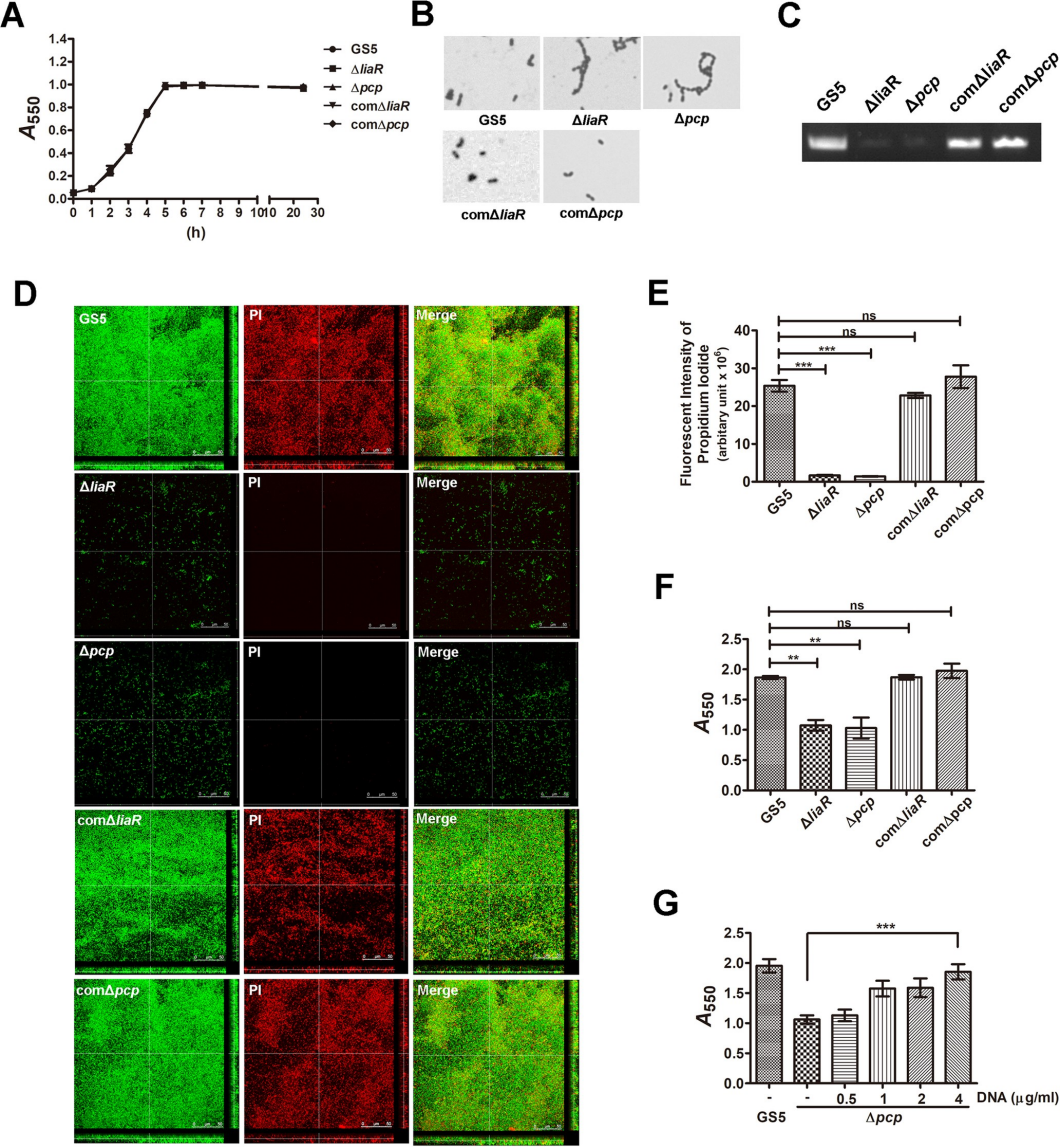
PCP mediates bacterial eDNA release contributing to *S*. *mutans* biofilm formation. (A) Growth curves of *S*. *mutans* GS5 wild type, Δ*liaR*, Δ*pcp*, comΔ*liaR* and comΔ*pcp* strains were detected by measuring the absorbance at 550 nm. (B) Light microscopic views of *S*. *mutans* GS5 wild type, Δ*liaR*, Δ*pcp*, comΔ*liaR*, and comΔ*pcp* cells. (C) eDNA released into the medium of *S*. *mutans* cultures, including wild type, Δ*liaR*, Δ*pcp*, comΔ*liaR*, and comΔ*pcp* was PCR-amplified by bacterial 16S rRNA primers, and the products were resolved on 1% agarose gels. (D) CLSM images represent *S*. *mutans* GS5 wild type, Δ*liaR*, Δ*pcp*, comΔ*liaR*, and comΔ*pcp* biofilms (630× magnification). *S*. *mutans* were labeled with GFP (green), and bacterial eDNA was stained with 10 μM propidium iodide. (E) The fluorescence intensity for the eDNA images of biofilm using propidium iodide staining was measured. The results show the quantified data of the extended focus images of biofilms using Image J software (National Institutes of Health). Data are expressed as the means ± standard deviations from three experiments. ***P < 0.001 by 1-way analysis of variance. ns, not significant. (F) Biofilms of *S*. *mutans* GS5 wild type, Δ*liaR*, Δ*pcp*, comΔ*liaR*, and comΔ*pcp* were stained with 0.1% crystal violet and the absorbance quantified at 550 nm. Data are expressed as the means ± standard deviations of triplicate experiments; **P < 0.01 by 1-way analysis of variance. ns, not significant. (G) Biofilms of *S*. *mutans* GS5 wild type and Δ*liaR* grown with or without series concentrations of purified bacterial eDNA were stained with 0.1% crystal violet. Staining was quantified by measuring the absorbance at 550 nm. Data are expressed as the means ± standard deviations of triplicate experiments; ***P < 0.001 by 1-way analysis of variance. These. experiments were repeated three times, with a representative experiment shown. GFP, green fluorescent protein; eDNA, extracellular deoxyribonucleic acid; PI, propidium iodide; and CLSM, confocal laser scanning microscopy.

**Fig 5:**
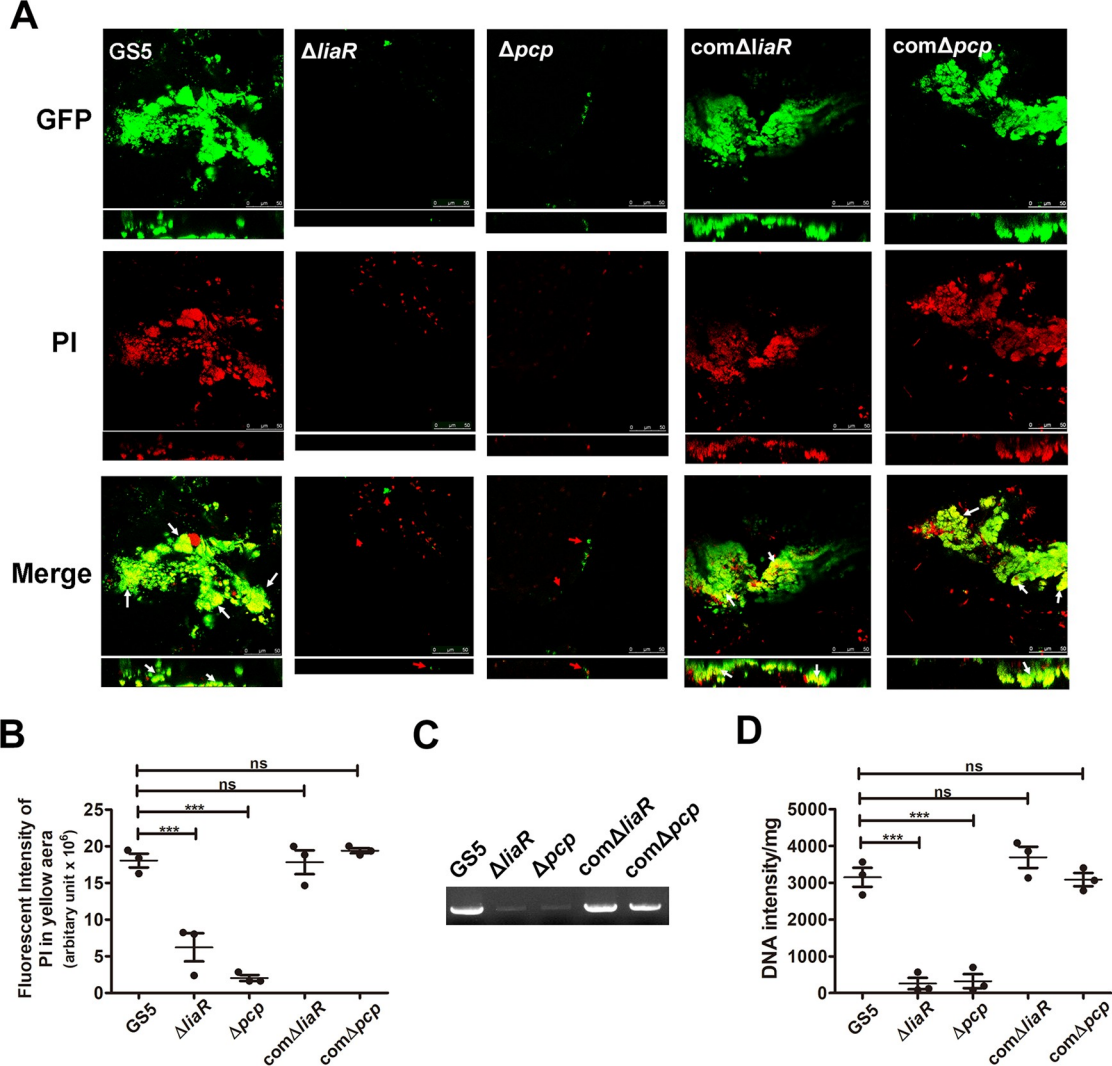
PCP mediates bacterial eDNA release contributing to *S*. *mutans* biofilm formation *in vivo*. (A) GFP-tagged *S*. *mutans* GS5 wild type, Δ*liaR*, Δ*pcp*, comΔ*liaR*, or comΔ*pcp* strains were intravenously injected into experimental IE rats, and the biofilm inside the vegetations harvested from injured heart valves were observed by CLSM (630× magnification). The data represent the CLSM images of biofilm formation inside the vegetation. *S*. *mutans* were labeled with GFP (green), and bacterial eDNA was stained with 10 μM propidium iodide. Yellow areas represent the presence of both *S*. *mutans* and eDNA (white arrows) and red arrows indicate single populations or aggregates without embedded eDNA. These experiments were replicated at least three times, with a representative experiment shown. (B) The fluorescence intensity of eDNA images using propidium iodide staining in the yellow aera was measured. The results show the quantified data of the extended focus images of biofilms using Image J software (National Institutes of Health). Scatter plots show values from individual rats, and each rat represents an independent experiment. Data are expressed as the means ± standard deviations. ***P < 0.001 by 1-way analysis of variance. (C and D) Total DNA isolated from vegetation (without lysing bacteria) was PCR-amplified with bacterial 16S rRNA primers and products were resolved on 1% agarose gels (C) and quantified with Image J software (National Institutes of Health) (D). Scatter plots show values from individual rats, and each rat represents an independent experiment. Data are expressed as the means ± standard deviations; ***P < 0.001 by 1-way analysis of variance. GFP, green fluorescent protein; IE, infective endocarditis; eDNA, extracellular deoxyribonucleic acid; and CLSM, confocal laser scanning microscopy; PI, propidium iodide.

Our previous data showed that bacterial eDNA release, mediated by autolysin (AtlA), contributes to *S*. *mutans* biofilm formation in IE [6]. Therefore, the role of PCP in eDNA-dependent biofilm formation was further investigated. Both of *liaR*- and *pcp*-deficient mutant strains showed normal growth rates but increased chain length compared with the wild type parental strain (Fig 4A and 4B). They also showed reduced ability to release DNA to the culture supernatant and reduced the eDNA-dependent biofilm formation capacity in sucrose-free defined M4 medium (Fig 4C–4F) (6). Complementation of bacterial DNA to the culture medium restored the biofilm formation ability of the *pcp*-deficient strain, confirming the role of eDNA in PCP-mediated biofilm formation (Fig 4G). Moreover, we further detected eDNA inside the vegetation by staining with propidium iodide (PI) in the rat IE model [6]. The confocal laser scanning microscopy (CLSM) image results showed that eDNA was embedded inside the bacterial aggregates of *S*. *mutans* GS5 and the complementation strains (Fig 5A, yellow areas, white arrows), but *liaR*- or *pcp*-deficient mutant strains appeared as single populations or aggregates without embedded eDNA (Fig 5A, red arrows). Quantification of the fluorescence intensity of eDNA images using PI staining in yellow areas showed that eDNA was reduced inside the mutant strain biofilms compared with wild-type the complementation strains (Fig 5B). To further confirm the reduced bacterial eDNA inside the vegetation samples of mutant strains, total DNA was extracted from the harvested vegetations without lysing the bacteria, and bacterial eDNA was semi-quantified by polymerase chain reaction (PCR) with bacterial 16S ribosomal RNA (rRNA) primers [6]. Consistent with the CLSM analysis, the eDNA levels detected in the *liaR*- or *pcp*-deficient mutant strains samples were much lower than in the wild-type and complementation strains (Fig 5C and 5D). Together, these data suggest that LiaR and PCP mediate bacteria to release eDNA, which contributes to bacterial biofilm formation in the pathogenesis of IE.

The phenotype of the *pcp*-deficient mutant strain is quite like the *atlA*-deficient mutant strain, in its increased cell length and reduced ability to release eDNA to biofilm *in vitro* and *in vivo* [6]. Therefore, AtlA expression and calcium ion-induced AtlA maturation were investigated [19]. However, neither the *liaR*- nor the *pcp*-deficient strains showed reduced ability to express and process AtlA (S4 Fig). These data suggest that PCP modulates *S*. *mutans* eDNA release without affecting AtlA expression and maturation.

### PCP mediates *S*. *mutans* biofilm formation via platelets in plasma *in vitro* and *in vivo*

**Fig 6:**
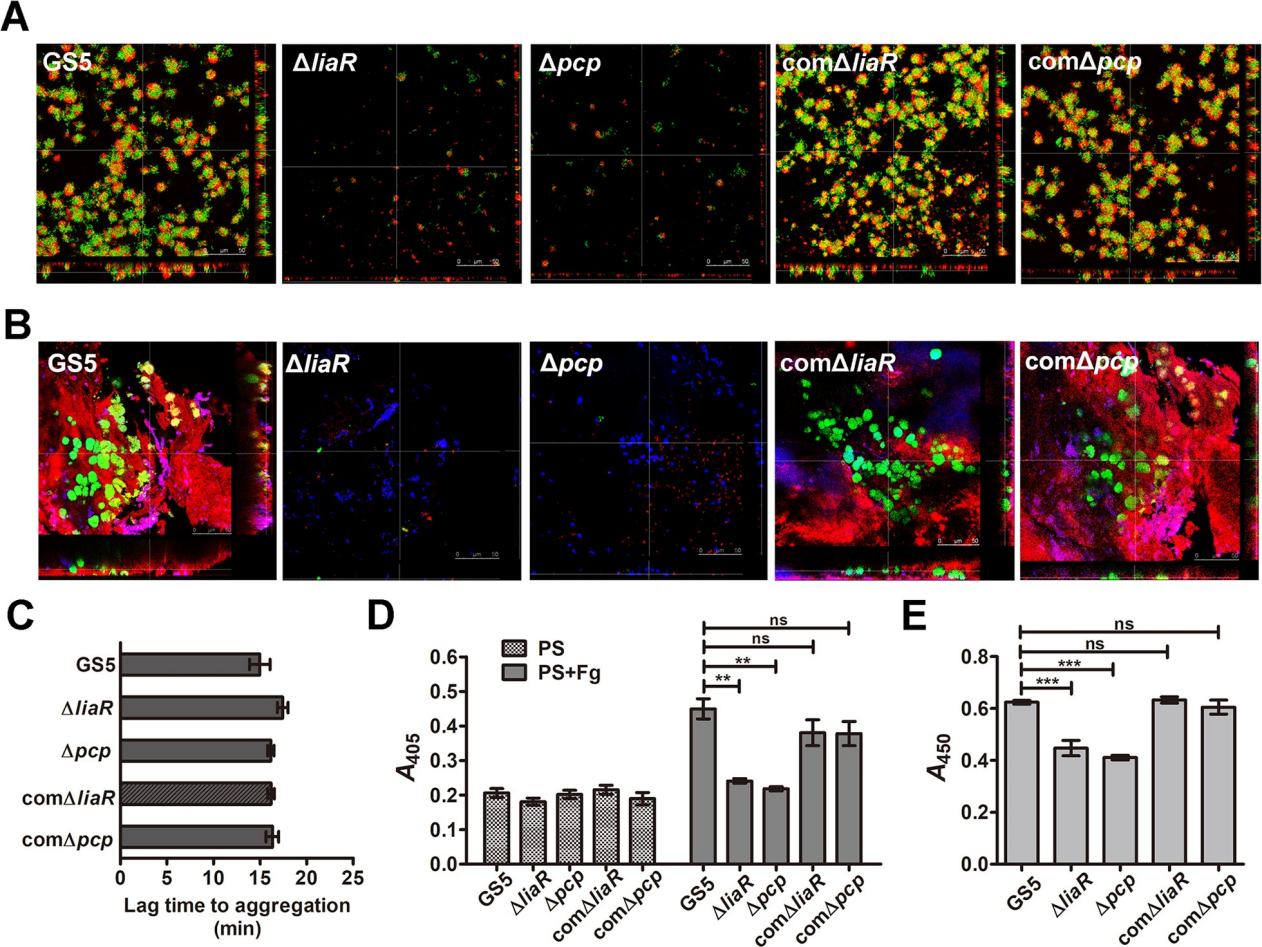
PCP mediates bacterial biofilm formation with platelets *in vitro* and *in vivo*. (A) Biofilms of *S*. *mutans* GS5 wild type, Δ*liaR*, Δ*pcp*, comΔ*liaR*, and comΔ*pcp* grown in PRP were observed by CLSM (magnification 630×). *S*. *mutans* were labeled with GFP (green), and platelets were stained with rhodamine-conjugated phalloidin (1:200 dilution). (B) Biofilms of *S*. *mutans* GS5 wild type, Δ*liaR*, Δ*pcp*, comΔ*liaR*, and comΔ*pcp* inside vegetation harvested from injured heart valves were observed by CLSM (630× magnification). *S*. *mutans* were labeled with GFP (green), and platelets were stained with an anti-CD42d antibody (1:50 dilution) followed by a rhodamine-conjugated secondary antibody (1:200 dilution). (C) Platelet aggregation was measured by a Lumi-Aggregometer. Bacterial ability to induce platelet aggregation was evaluated by detecting the lag time, which indicates the time between the addition of bacterium to the PRP suspensions and onset of aggregation response. Data are expressed as the means ± standard deviations of triplicate experiments. (D) Microtiter plate wells were coated with *S*. *mutans* GS5 wild type, Δ*liaR*, Δ*pcp*, comΔ*liaR*, or comΔ*pcp* strains then platelet suspensions with or without 100 μg/mL Fg were added. Platelet adherence was measured by acid phosphatase activity (OD_405_) as described in the Materials and Methods. Data are expressed as the means ± standard deviations of triplicate experiments; **P < 0.01 by 1-way analysis of variance. ns, not significant. (E) Binding capacity to immobilized Fg were compared. Microtiter plate wells were coated with Fg, and bacteria suspensions (*S*. *mutans* GS5 wild type, *ΔliaR*, *Δpcp*, comΔ*liaR*, or *comΔpcp*) were added. The adhered bacteria were measured as described in the Materials and Methods. Data are expressed as the means ± standard deviations of triplicate experiments; ***P < 0.001 by 1-way analysis of variance. ns, not significant. These experiments were replicated thrice, with a representative experiment shown. PRP, platelet rich plasma; GFP, green fluorescent protein; CLSM, confocal laser scanning microscopy; IE, infective endocarditis; Fg, Fibrinogen; and PS, platelet suspension.

Bacteria bind to platelets and form aggregates to enhance bacterial biofilm formation in IE [3]; therefore, the role of PCP in platelet-dependent bacterial biofilm formation was investigated further. Both *liaR*- and *pcp*-deficient mutant strains showed reduced ability to form biofilm with platelets in the plasma *in vitro* (Fig 6A). Consistently, they also showed reduced ability to form biofilms with platelets on damaged valves in experimental IE rat model (Fig 6B). Bacterial abilities to bind platelets and induce their aggregation mediates bacterial ability to form biofilms with platelets [3]. Interestingly, neither the *liaR*- nor the *pcp*-deficient strains showed reduced ability to induce platelet aggregation (Fig 6C) but rather had a lower ability to bind platelets when fibrinogen (Fg) was added compared with the parental strain and complementation strains (Fig 6D). The *liaR*- and *pcp*-deficient mutant strains showed a reduced ability to adhere to immobilized Fg, suggesting that the *pcp*-deficient strain has reduced ability to bind platelets, which is at least partly due to the reduced Fg binding ability (Fig 6E). Together, these data suggest that PCP is an essential gene for *S*. *mutans* biofilm formation *in vivo* as it controls eDNA release and bacterial platelet adhesion on damaged heart valves to induce IE.

## Discussion

Using an *in vivo* survival assay, the present study identified an important *S*. *mutans* response regulator, LiaR, which regulates bacterial biofilm formation on the damaged heart valve in experimental rat IE model. Distinct from other identified biofilm-regulating TCSs of *S*. *mutans*, which regulate Gtf-mediated glucan formation, LiaR regulates bacterial eDNA release and platelet adhesion *in vivo*. In addition to the response regulators, we also tried to identify the sensor kinases involved in *pcp* regulation and bacterial biofilm formation on the damaged heart valve, and we found none of the tested sensor kinases (including SMU.1128, SMU.1145, SMU.1814, SMU.1037, SMU.1009, SMU.1965, SMU.577, SMU.486 [LiaS], and SMU.1548) were found to be involved in *pcp* regulation and bacterial biofilm formation *in vivo*, including LiaS (S5 Fig). These data suggest that other sensor kinases or more than two in *S*. *mutans* are responsible for regulating PCP expression and biofilm formation in IE. Consistent with the present results, Chong *et al*. also reported different roles for LiaS and LiaR in the regulation of *S*. *mutans* virulence factor expression, such as *gbpC* expression and mutacin IV production [20]. Therefore, the sensor kinase responsible for regulating PCP expression and biofilm formation on heart valves in IE needs further examination.

In addition to LiaR, the current *in vivo* survival assay results also revealed the SMU.1146-deficient strain had reduced ability to colonize damaged heart valves *in vivo*, compared with other mutant strains (Fig 1B). Our preliminary data showed SMU.1146 deficiency did not significantly reduce the ability to induce vegetation formation *in vivo*, but the role of SMU.1146 in the pathogenesis of IE require further investigation. While the roles of other *S*. *mutans* response regulators [SMU.1517 (VicR), SMU.1129 (CiaR), SMU.1917 (ComE), and GcrR (CovR)] in the pathogenesis of IE were not investigated here, previous studies have demonstrated their important roles in the regulation of bacterial virulence gene expression in oral streptococci, including *S*. *mutans* [21]. Previously, we demonstrated the role of VicK in regulation of AtlA maturation, which plays important role in bacterial eDNA-dependent biofilm formation in IE [6]. However, because VicR is essential for bacterial viability, *vicR*-deficient strains could not be generated to investigate the role of VicR in AtlA maturation and *S*. *mutans* biofilm formation in IE. Therefore, the roles of VicR, GcrR, CiaR, and ComE in the pathogenesis of IE needs further investigation.

In addition to regulate *S*. *mutans* biofilm formation on damaged valves, LiaR has also been shown to play a role in the formation of biofilm under oxidative stress, development of genetic competence, and resistance to acidic pH [12,22]. It has also been reported to regulate expression of numerous genes, including *pcp* (SMU.753) [23]. However, no study has yet characterized the function of PCP in the modulation of *S*. *mutans* virulence in bacterial pathogenesis. PCP is a small protein composed of only 99 amino acids that contains a domain of PspC which is a member of the Psp stress response system [24]. The main function of the Psp system is to protect bacteria from several extreme stresses, which has been well-characterized in Gram-negative bacteria, including *E*. *coli* and *Y*. *enterocolitica* [25]. The Psp system in Gram-negative bacteria contains four core components: PspF, -A, -B, and -C [24,26]. PspA forms a complex with PspF to inhibit its activity and can also interact with the PspB-PspC complex in the inner membrane, where it may assist bacterial stress relief and maintains the proton motive force [27,28]. PspB and PspC form an integral inner membrane complex involved in activation of the Psp response and prevents toxicity of mislocalized secretins in *Y*. *enterocolitica* strains [29,30]. Most review manuscripts indicate the crucial role of PspA in the Psp stress response system [24,31]. However, *pspC*-null, but not *pspA*-null, strain of *Y*. *enterocolitica* shows severely attenuated virulence in a mouse infection model [32], suggesting the independent role of PspC in bacterial virulence modulation. In addition, PspA homologs are only found in some Gram-positive bacteria, including *Bacillus subtilis* [33] and *Mycobacterium tuberculosis* [34], but PCP can be found in most streptococci (https://www.ncbi.nlm.nih.gov/gene). Although PspC has been reported to have a role in preventing the toxicity of mislocalized secretins in *Y*. *enterocolitica* strains [29,30], little is known about the actions of PspC and the function of the PspC domain. PCP is a protein family with relatively low molecular weight and lacks any other identified functional domains, suggesting it may influence bacterial eDNA release and platelet adhesion by modulating the expression or function of other bacterial surface proteins in *S*. *mutans*. Therefore, our current working hypothesis is that PspC or PCP may affect the function and/or stability of bacterial surface-located proteins or outer membrane instability in some Gram-negative bacteria. The function of PCP will be further investigated, which may also provide important information on the function of PspC in Gram-negative bacteria.

Our previous study demonstrated that bacterial eDNA contributes to *S*. *mutans* biofilm formation on damaged heart valves of endocarditis rats [6]. The same study also showed that the autolysis protein AtlA in *S*. *mutans* contributes to bacterial eDNA release to biofilm *in vitro* and *in vivo* [6]. The phenotype of the *pcp*-deficient strain is quite similar to that of the *atlA*-deficient strain in its increased chain length and reduced ability to release eDNA (Fig 4), suggesting that PCP may play a role in AtlA-mediated autolysis. However, AtlA expression and calcium ion-induced AtlA maturation remain unchanged in *pcp*-deficient strain compared with the wild type strain (S1 Fig). Similarly, a previous study also showed inactivation of *S*. *mutans* chaperones RopA and PrsA increased bacterial chain length but did not influence AtlA expression [35]. These data imply that PCP may modulate the function of AtlA or influence *S*. *mutans* autolysis systems. In addition to AtlA-mediated autolysis, an *S*. *mutans* eDNA release system involving protein secretion machinery-mediated vesicle formation has also been proposed [36], but its role in the pathogenesis of IE and the underlying mechanism remain unclear. Therefore, the role of PCP in protein secretion machinery-mediated eDNA release will be also further investigated. A recent study showed that eDNA crosslinks glucan to stabilize the *S*. *mutans* biofilm on dental surface [37]. However, our previous study showed that glucosyltransferase-deficient strains do not have a reduction in the ability to cause IE [16], suggesting that glucan may not play a role in *S*. *mutans* biofilm formation on damaged heart valves. Moreover, the *pcp*-deficient mutant strain had no defect in the expression of glucosyltransferases or in glucan-dependent biofilm formation (S6 Fig). These data further confirm that glucan does not play a major role for bacterial biofilm formation during the pathogenesis of IE.

In addition to mediating eDNA release, PCP also mediates bacterial ability to bind platelets and form biofilm with them in an Fg-dependent manner. Fg-binding proteins, such as ClfA in *Staphylococcus aureus*, have been identified in several IE pathogens and play important roles in bacterial binding to platelets and IE pathogenesis [38,39]. However, no Fg-binding protein was identified in *S*. *mutans*. Here, addition of Fg enhanced *S*. *mutans* binding to platelets, suggesting an unidentified Fg-binding protein plays important roles in *S*. *mutans* biofilm formation with platelets. It is posited that other *S*. *mutans* Fg-binding protein(s) will be identified by investigating the function of PCP further. In addition, our previous study showed bacterial biofilm with platelets on damaged valves recruits neutrophils and induces NET formation, which contributes to vegetation expansion. Therefore, the *liaR*- or *pcp*-deficient strains, which have a reduced ability to form biofilms with platelets on damaged valves, also show a reduced ability to induce NETs (Fig 6B), which partly contributes to the reduced vegetation formation in the IE rat models (Figs 1D and 2E).

Taken together, the present study identified an *S*. *mutans* response regulator (LiaR) and downstream gene (PCP) which play roles in *S*. *mutans* biofilm formation on damaged heart valves in the pathogenesis of IE. These data also confirm the crucial roles of bacterial eDNA release and platelet adhesion in the pathogenesis of IE. In addition, conservation of PCP among streptococci further suggest the putative role of the PCP in biofilm formation of other streptococci.

## Materials and methods

### Ethics statement

Human blood samples were collected from healthy volunteers, and the study was approved by the National Taiwan University Hospital Committee for Regulation of Human Specimens and Volunteers (Taipei, Taiwan; No. 201901005RINC). All human volunteers provided informed consent.

### Bacterial strains and plasmids

*S*. *mutans* UA159, GS5, and isogenic deletion-mutant strains were grown and maintained in brain-heart infusion broth (Difco Laboratories Inc., Detroit, MI, USA). The insertion mutants of response transcription regulators of *S*. *mutans* UA159, including SMU.1146, SMU.927, SMU.1815, SMU.659, SMU.1038, SMU.1008, SMU.1964, SMU.576, SMU.487, and SMU.1547, were kindly provided by Dr. Li [10]. GS5 *liaR*-deficient (Δ*liaR*), *pcp*-deficient (Δ*pcp*), and *pcp*-complemented (comΔ*pcp*) strain construction is described below. GFP-tagged bacteria were generated by transformation with a shuttle plasmid (pPDGFPuv) containing the *GFPuv* sequence described in our previous study [19]. For selection of colonies after transformation, the growth medium was supplemented with erythromycin (5 μg/mL; insertion mutants of transcription regulators), kanamycin (500 μg/mL; Δ*liaR*, Δ*pcp*, comΔ*liaR* and comΔ*pcp*), chloramphenicol (5 μg/mL; comΔliaR and comΔ*pcp*), or spectinomycin (500 μg/mL; pPDGFPuv).

### Δ*lia*R, Δ*pcp*, and comΔ*pcp* strain construction

All references to genomic loci are based on the *S*. *mutans* UA159 genome database (https://www.ncbi.nlm.nih.gov/genome/). To construct Δ*liaR* and Δ*pcp* strains, these genes in *S*. *mutans* GS5 were disrupted by insertion of a promoterless kanamycin cassette using a ligation-PCR mutagenesis strategy [40]. Briefly, the promoterless kanamycin resistance gene fragment was isolated from the pALH124 plasmid by *Eco*RI digestion [41]. Approximately 500-bp fragments before and/or after *liaR* and *pcp* were PCR-amplified using the forward and reverse primers listed in Table 1. PCR reaction products were digested with *Eco*RI, ligated with the kanamycin resistance gene fragment, and transformed directly into *S*. *mutans* GS5. Correct allelic replacement in the derived isogenic mutant strains was confirmed by PCR amplification and RT-PCR analysis was performed to verify the loss of *liaR* and *pcp* expression and confirm expression of genes downstream of *liaR* and *pcp*. For construction of the comΔ*liaR* and comΔ*pcp* strains, gene fragments of the *liaR* promoter, *liaR*, *pcp* promoter, and *pcp* were PCR-amplified using the primers listed in Table 1. PCR products were digested with *Xba*I or *EcoR*I and cloned into the pMC340B_Cm plasmid that contained the chloramphenicol resistance gene [19]. The resulting plasmid was transformed into the Δ*liaR* or Δ*pcp* strains to generate the comΔ*liaR* or comΔ*pcp* strains. Expression of *liaR* or *pcp* was further confirmed by RT-PCR.

### *S*. *mutans*-induced IE rat model and *in vivo* survival assay

Approval of animal use was obtained from the National Taiwan University Institutional Animal Care and Use Committee (Taipei, Taiwan, Republic of China) prior to initiation of experiments. A modified rat model of experimental streptococcal endocarditis was performed as previously described [16]. Briefly, a polyethylene tube with a stainless-steel wire embedded inside was inserted into the left carotid artery above the clavicles and advanced toward the chest to create injury at the aortic valves. Twenty-four hours after catheter placement, rats were intravenously infected with the bacteria at 1 × 10^9^ CFU. For *in vivo* survival assay, a mix of 10 insertion mutants of transcription regulators was injected (10^8^ for each). At 24 h post-infection, the vegetation was harvested and homogenized by ultrasonication. The bacteria colonized inside the vegetation was detected by PCR using specific primers listed in Table 1. For confocal laser scanning microscopy (CLSM) analysis, GFP-tagged *S*. *mutans* GS5 wild type, Δ*liaR*, Δ*pcp*, or comΔ*pcp* strains (1 × 10^9^ CFU) were intravenously injected into the tail veins of Wistar rats. For the detection of bacterial eDNA, the vegetation was stained with propidium iodide. For platelets, the vegetation was stained with an anti-CD42d antibody (1:50 dilution; eBioscience, San Diego, CA, USA), followed by Texas red-tagged anti-rabbit immunoglobulin G antibody (1:500 dilution; Jackson ImmunoResearch Labs, West Grove, PA, USA), and DNA was stained with Hoechst 33258 (Sigma-Aldrich, St. Louis, MO, USA). Bacterial biofilms were then observed on a confocal microscope (Leica TCS SP5). For histologic analyses and gram staining, heart tissue was fixed with 2% paraformaldehyde, embedded in paraffin, cut into 3-μm-thick slices, and stained with hematoxylin and eosin or modified gram staining [42].

### Microarray analysis

The microarray analysis of GS5 wild type and Δ*liaR* strains was performed as previously described [19]. *S*. *mutans* oligonucleotide microarrays were provided by The Institute for Genomic Research and supported by the National Institute of Dental and Craniofacial Research (NIH, USA). RNA of the bacteria grown in the exponential phase was extracted using an RNeasy Mini Kit (Qiagen, Germantown, MD, USA) following the manufacturer’s protocol. After purification of RNA using an RNA cleanup kit (Qiagen), cDNA synthesis, Cy dye coupling, and array hybridization was performed as The Institute for Genomic Research. Briefly, cDNA synthesis was carried out with random hexamers and RT, and the mixture was incubated at 42°C for 16 h. Following completion of cDNA synthesis, RNA was hydrolyzed with NaOH and the aminoallyl-labeled cDNA samples were purified using a QIAquick purification kit (Qiagen) followed by drying in a speed vacuum. The samples were then resuspended in 1 M NaCO_3_ and incubated with Cy dye (resuspended as recommended by the manufacturer) for 2 h in the dark. Each Cy3-labeled experimental sample was mixed with a Cy5-labeled reference sample and the mixtures were dried using a speed vacuum. For hybridization, the samples were resuspended in hybridization buffer and heated at 90°C for 10 min. Following a quick centrifugation, the Cy dye-coupled probes were applied to microarray slides and incubated at 42°C water bath for 17 h. Slides were then washed and scanned using an Axon GenePix 4000B scanner (Axon Instruments, Foster City, CA, USA). The data were analyzed by GeneSpring software and Significance Analysis of Oral Pathogen Microarray Data [43]. Microarray analysis data were further confirmed by RT-PCR, which was performed as previously described [44]. The primers for RT-PCR are listed in Table 1.

### Recombinant LiaR construction and anti-LiaR antibody preparation

Recombinant LiaR expression plasmids were constructed by cloning of relevant PCR amplification products into the *Bam*HI site on the pQE30 vector (Qiagen) containing a sequence encoding an N-terminal in-frame 6XHis-tag. Th PCR primers for generation of LiaR are listed in Table 1. For expression, resultant LiaR expression plasmids were transformed into *E*. *coli* M15, and protein expression was induced during logarithmic growth by addition of 1 mM isopropyl-β-d-thiogalactopyranoside. After induction, the proteins were purified via nickel chelating affinity chromatography (Qiagen) as previously described [45]. The purity of the proteins was analyzed by Coomassie blue staining following sodium dodecyl sulfate (SDS)-polyacrylamide gel electrophoresis analysis. Antisera against LiaR were prepared as previously described [46]. Briefly, the purified rLiaR was suspended in 3 mL of phosphate-buffered saline (PBS) and emulsified in Freund’s complete adjuvant before being used to immunize rats by intracutaneous injection. Two subsequent booster injections were intravenously given at 2-week intervals using incomplete Freund’s adjuvant, and sera was collected 2 d after the last boost. The specificity of the anti-LaR rat serum was confirmed by Western blot analysis using total protein extracts from wild type *S*. *mutans* or the *liaR*-deficient mutant strain.

### ChIP assay

ChIP assays were performed with the anti-LiaR antibody as previously described [47] with some modifications. Briefly, *S*. *mutans* GS5 were cultured to exponential phase (OD_550_ = 0.4), then formaldehyde at a final concentration of 1% was added directly to the culture. The cross-linking reaction mixture was incubated at room temperature for 15 min with continuous shaking. Then, the reaction was stopped by addition of glycine (to 125 mM) for 5 min at room temperature. After washing with PBS three times by centrifugation, the chilled cells were resuspended in lysis buffer [50 mM Tris-HCl (pH 8.0), 500 mM NaCl, 1 mM EDTA, 0.1% Triton X-100, 0.1% SDS, 0.1% sodium deoxycholate, and 100 mM PMSF] containing glass beads (BioSpec, Bartlesville, OK, USA). The bacterial cells were sonicated (FastPrep FP120 homogenizer, Qbiogene Inc., Montreal, QC, Canada) to fragment chromosomal DNA to a majority size range of 0.5–1.0 kb. After centrifugation, the supernatant was harvested and used as the “input” fraction for the ChIP assay. The anti-LiaR or preimmune rat serum (5 μL/mL) was added to the input fraction for immunoprecipitation. After incubation overnight at 4 ^o^C on a rotating platform, protein A Sepharose beads (Amersham Pharmacia Biotech, Piscataway, NJ, USA) were added to the mixtures and incubated for 1 h. After washing with lysis buffer twice, the beads were sequentially washed twice by ChIP wash buffer [10 mM Tris-HCl (pH 8.0), 1 mM EDTA, 0.25 M LiCl, and 0.5% sodium deoxycholate] and twice with TE buffer [10 mM Tris-HCl (pH 8.0) and 1 mM EDTA]. The antibody-protein-DNA complexes were further eluted with elution buffer [1% SDS, TE (pH 8.0), and 10 mM dithiothreitol] for 30 min at 37 ^o^C. After protein digestion by proteinase K (20 mg/mL; Invitrogen, Carlsbad, CA, USA), the DNA were further purified by addition of equal volumes of a phenol-chloroform-isoamyl alcohol mix (25:24:1). After vortexing, the mixture was centrifuged at 4°C for 5 min at 16,000 × *g*. The aqueous phase was harvested, and the DNA was further precipitated by addition of 0.1 volumes of 3 M sodium acetate and 2.5 volumes of ice-cold 100% ethanol and incubated at -80°C. After centrifugation, the precipitated sample was air dried and suspended in 30 μL of TE buffer, which was used as the “output” fraction for PCR analyses.

### Semiquantification of eDNA

Bacterial eDNA was detected and purified as previously described [6]. Briefly, the biofilms adhered to the culture tubes were disrupted and suspended by violent agitation. The culture medium was harvested by centrifugation. 1 μL of supernatant was used as a template for semiquantitative PCR (25 cycles) using specific bacterial 16S rRNA primers (Table 1). M4 medium (1 μL) without seeding bacteria was used as a negative control, and medium containing *S*. *mutans* GS5 genomic DNA was used as a positive PCR control. For the detection of bacterial eDNA inside the vegetation, total DNA was extracted without lysing bacteria using the Gentra Puregene Kit (Qiagen) following the manufacturer’s protocol. The DNA pellet was hydrated in 100 μL DNA hydration solution, and 1 μL was used as a template for semiquantitative PCR (35 cycles) using specific bacterial 16S rRNA primers (Table 1).

### Preparation of human platelets

Human blood samples were collected from healthy volunteers and added to 3.2% sodium citrate at a ratio of 9:1 by volume. Human platelet-rich plasma (PRP) was prepared by centrifugation as previously described [48]. The washed platelet suspension was also prepared according to the protocol previously described [3,48,49]. PRP was supplemented with prostaglandin E1 (0.5 μM) and heparin (6.4 IU/mL) to stabilize platelets. After centrifugation, the platelets were washed twice with Tyrode solution (136.8 mM NaCl, 2.8 mM KCl, 11.9 mM NaHCO_3_, 1.1 mM MgCl_2_, 0.33 mM NaH_2_PO_4_, 1.0 mM CaCl_2_, 11.2 mM glucose, and 3.5 mg/mL bovine serum albumin [BSA]) with prostaglandin E1 (0.5 μM) and heparin (6.4 IU/mL). The preparation was finally resuspended in Tyrode solution at a concentration of 3–5 × 10^8^ platelets/mL.

### Biofilm formation assay

The eDNA-dependent biofilm assay was performed as previously described using sucrose-free defined M4 medium [6]. Briefly, bacterial biofilm growth was initiated by inoculating individual wells of a 96-well polystyrene microtiter plate with approximately 10^7^ CFU in 200 μL M4 medium. Each assay was performed in triplicate, and wells without biofilms were used as blank controls. After a 16-h incubation at 37°C, the biofilm were stained with 0.1% crystal violet and quantified by measuring the absorbance at 550 nm using a MicroELISA reader (Dynatech Corp., Alexandria, VA, USA). For CLSM analysis, *GFPuv*-tagged strains were transformed with pPDGFPuv, and the bacterial biofilms were cultured in a 24-well plate with a round glass coverslip in individual wells. For eDNA-dependent biofilm, the biofilms were cultured in M4 medium. For bacteria-platelet aggregate biofilm, the biofilms were grown in the PRP [3]. After a 16-h incubation, biofilms that had formed on the glass coverslip were gently washed with PBS three times and then fixed with 2% paraformadehyde for 15 min. After fixation, bacterial eDNA embedded in the eDNA-dependent biofilm was stained with 10 μM propidium iodide, and the platelets in the bacteria-platelet aggregate biofilm were stained with rhodamine-conjugated phalloidin (1:200 dilution; Invitrogen). After washing with PBS three times, the coverslips were transferred to a slide and observed by CLSM (Leica TCS SP5).

### Platelet adhesion assay

The platelet adhesion assay was performed as previously described with some modifications [50]. Microtiter plate wells were coated with 100 μL of bacteria (OD_550_ = 1.0); fibrinogen (20 μg/mL) and BSA (1%) coated wells served as positive and negative controls, respectively. After blocking with 1% BSA at 37°C for 1 h, the plate was washed with Tyrode solution three times, 50 μL of platelet suspension (3–5 × 10^8^ platelets/mL) with or without Fg (100 μg/mL) was added and incubated at 37°C for 30 min. Pure Fg was purchased from Sigma-Aldrich. Nonadherent platelets were removed by three washes with Tyrode solution, and bound platelets were lysed at 37°C for 2 h in acid phosphatase detection buffer [0.1 M sodium acetate (pH 5.5)], 0.1% Triton X-100, and 10 mM *p*-nitrophenol phosphate]. The results were quantified by measuring the absorbance at 405 nm using a MicroELISA reader.

### Bacterial Fg-adherence assay

The Fg adherence assay was performed as previously described [46]. Briefly, the microtiter plates were coated with 50 μL of purified Fg (0.1 mg/mL in 0.05 M sodium carbonate buffer, pH 9.6). After washing three times with PBS-Tris buffer, plates were blocked with 1% BSA. Overnight-cultured *S*. *mutans* wild type and mutant strains were washed, resuspended in 0.5 mL of PBS, and adjusted to an absorbance of 0.9 at 550 nm. The bacterial suspension (50 μL/well) was then applied to the Fg-coated assay plates and incubated 37°C at room temperature for 2 h. Nonadherent bacteria were removed with three gentle washes of PBS. Adherent bacteria were fixed with a 15-min incubation at 60°C. After cooling to room temperature, a 1:5000 dilution of antiserum against *S*. *mutans* [51] was added, followed by incubation for 1 h at 37°C. After washing with PBS three times, a 1:10,000 dilution of horseradish peroxidase-labeled goat anti-rabbit immunoglobulin G (Sigma) was applied, followed by addition of 3,3’,5,5’-tetramethylbenzidine substrate (Clinical Science Product Inc. MA, USA). The reactions were stopped by addition of 2 N H_2_SO_4_, and the absorbance at 450 nm was measured using a MicroELISA reader.

### Statistical analysis

An unpaired, 2-tailed Student’s *t*-test was used to analyze the statistical significance of the difference between two sets of data. Differences between more than two sets of data were assessed using 1-way analysis of variance followed by the Bonferroni multiple-comparisons test. The Kruskal-Wallis test with subsequent Dunn’s test were used to analyze nonparametrically distributed data. A *P* < 0.05 was considered statistically significant.

## Supporting information

S1 Table Microarray analysis of genes differentially expressed in *liaR*-deficient mutant versus wild type strains. The genes with at least a twofold change in expression and p<0.05 are listed.
(DOCX)

S1 Fig Vegetation on the heart valve in the rat experimental IE model. *S*. *mutans* GS5 wild-type, Δ*liaR*, Δ*pcp*, comΔ*liaR*, or comΔ*pcp* strains were intravenously injected into experimental IE rat models. Photographs of the vegetation formation on the heat valve are shown. The markers represent the valves (dash lines) and vegetations (black arrows). Scale bars represent 1 mm.
(TIF)

S2 Fig Histological analysis of vegetation formation in the rat experimental IE model. *S*. *mutans* GS5 wild-type, Δ*liaR*, Δ*pcp*, comΔ*liaR*, or comΔ*pcp* strains were intravenously injected into experimental IE rat models and the rats were sacrificed 24 h post-infection. Heart tissue was then collected for histopathology staining. The markers represent the valves (solid lines) and the vegetations (dash circles). Scale bars represent 200 μm.
(TIF)

S3 Fig Bacterial colonization inside the vegetation. *S*. *mutans* GS5 wild-type, Δ*liaR*, Δ*pcp*, comΔ*liaR*, or comΔpcp strains were intravenously injected into experimental IE rat models and the rats were sacrificed 24 h post-infection. Vegetation was subsequently collected for gram staining. The markers represent the vegetations (dash circle) and the bacteria (black arrows). The bacteria cannot be easily detected by gram staining in the Δ*liaR* and Δ*pcp* samples. Scale bars represent 100 μm (left panels) and 20 μm (right panels).
(TIF)

S4 Fig PCP has no effect on AtlA expression and maturation. *S*. *mutans* GS5 wild type, Δ*liaR*, Δ*pcp*, *comΔliaR* and *comΔpcp* were cultured in BHI medium in the absence (lane 1) or presence (lanes 2) of the indicated concentrations of CaCl_2_, and the bacterial surface proteins were extracted by 4% SDS. AtlA expression and the mature form of AtlA (90 kDa) were detected by Western blot analysis.
(TIF)

S5 Fig None of the tested sensor kinases were found to be involved in *pcp* regulation and bacterial biofilm formation *in vivo*. (A) Identification of the sensor kinase involved in bacterial biofilm formation in *in vivo* survival assay. The upper panel is a representative PCR result of injected bacterial mixtures (input PCR); the bottom panel is a representative PCR result of bacteria colonized inside the vegetation. M, DNA marker; lane 1, the PCR result using the primers for detecting *SMU*.*1128*-deficient mutant; lane 2, *SMU*.*1145*; lane 3, *SMU*.*1814*; lane 4, *SMU*.*1037*; lane 5, *SMU*.*1009*; lane 6, *SMU*.*1965*; lane 7, *SMU*.*577*; lane 8, *SMU*.*486* (*liaS*); lane 9, *SMU*.*1548*; and PC, positive control, PCR product of 16S rRNA gene. (B) Surface protein of *S*. *mutans* UA159 wild type and the isogenic mutant strains of sensor kinases were extracted by 4% SDS, and the expression of PCP were detected by Western blot analysis.
(TIF)

S6 Fig The *pcp*-deficient mutant strain had no defect in the expression of glucosyltransferases or in glucan-dependent biofilm formation. (A) Surface protein of *S*. *mutans* GS5 wild type, Δ*pcp* and comΔ*pcp* were extracted by 4% SDS, and the expression of glucosyltransferase-I (GtfB) and glucan binding protein B (GbpB) were detected by Western blot analysis. (B) Biofilms of *S*. *mutans* GS5 wild type, Δ*pcp*, and comΔ*pcp* cultured in BHI medium containing 1% sucrose were stained with 0.1% crystal violet and the absorbance quantified at 550 nm. Data are expressed as the means ± standard deviations of triplicate experiments; ns, not significant by 1-way analysis of variance.
(TIF)
