# Supplementary material for: PspC domain-containing protein (PCP) determines Streptococcus mutans biofilm formation through bacterial extracellular DNA release and platelet adhesion in experimental endocarditis
Source: PLoS Pathog. 2021 Feb 12;17(2):e1009289. doi: 10.1371/journal.ppat.1009289 (PMC7906467; doi:10.1371/journal.ppat.1009289)
Supplement: S1 Table — The genes with at least a twofold change in expression and p<0.05 are listed. (DOCX) [file ppat.1009289.s001.docx]

**S1 Table. Microarray analysis of genes differentially expressed in *liaR*-deficient mutant versus wild type strains.** The genes with at least a twofold change in expression and p<0.05 are listed.

| **Genbank Tag** | **Description** | **n-Fold difference between RR11 mutant/WT strain** | **p-value** |
| --- | --- | --- | --- |
| SMU.753 | conserved hypothetical protein | 0.0862 | 5.18E-06 |
| SMU.139 | conserved hypothetical protein | 0.259 | 0.0279 |
| SMU.140 | putative glutathione reductase | 0.275 | 2.79E-04 |
| SMU.141 | conserved hypothetical protein | 0.324 | 8.13E-04 |
| SMU.1257c | conserved hypothetical protein | 0.46 | 8.65E-03 |
| SMU.1904c | hypothetical protein | 0.461 | 9.2E-03 |
| SMU.1056 | hypothetical protein | 0.483 | 0.0371 |
| SMU.1955 | putative co-chaperonin GroES | 2.021 | 1.54E-03 |
| SMU.662 | conserved hypothetical protein possible membrane protein | 2.174 | 3.04E-02 |
| SMU.239c | hypothetical protein | 2.227 | 4.69E-02 |
| SMU.148 | putative alcohol-acetaldehyde dehydrogenase | 2.256 | 2.88E-03 |
| SMU.2133c | putative membrane protein | 2.478 | 0.0617 |
| SMU.173 | putative ppGpp-regulated growth inhibitor | 2.56 | 8.77E-03 |
| SMU.172 | conserved hypothetical protein putative cell growth regulatory | 2.644 | 4.37E-04 |
| SMU.1094 | putative ABC transporter, ATP-binding protein | 3.283 | 0.0187 |
| SMU.1067c | putative ABC transporter, permease protein | 3.288 | 7.96E-04 |
| SMU.83 | heat shock protein DnaJ (HSP-40) | 3.342 | 7.82E-04 |
| SMU.932 | hypothetical protein | 3.63 | 0.0192 |
| SMU.82 | heat shock protein, DnaK (HSP-70) | 3.897 | 1.32E-05 |
| SMU.1070c | conserved hypothetical protein | 3.975 | 4.1E-04 |
| SMU.80 | transcriptional regulator repressor (HrcA) of class I | 4.759 | 8.88E-04 |
| SMU.1855 | hypothetical protein | 4.993 | 3.15E-05 |
| SMU.1854 | conserved hypothetical protein | 5.319 | 2.86E-04 |
| SMU.81 | heat shock protein GrpE (HSP-70 cofactor) | 5.547 | 5.69E-06 |
| SMU.432 | putative ABC transporter, integral membrane protein | 5.745 | 0.0262 |
