# Supplementary figures and images for: PspC domain-containing protein (PCP) determines Streptococcus mutans biofilm formation through bacterial extracellular DNA release and platelet adhesion in experimental endocarditis
Source: PLoS Pathog. 2021 Feb 12;17(2):e1009289. doi: 10.1371/journal.ppat.1009289 (PMC7906467; doi:10.1371/journal.ppat.1009289)

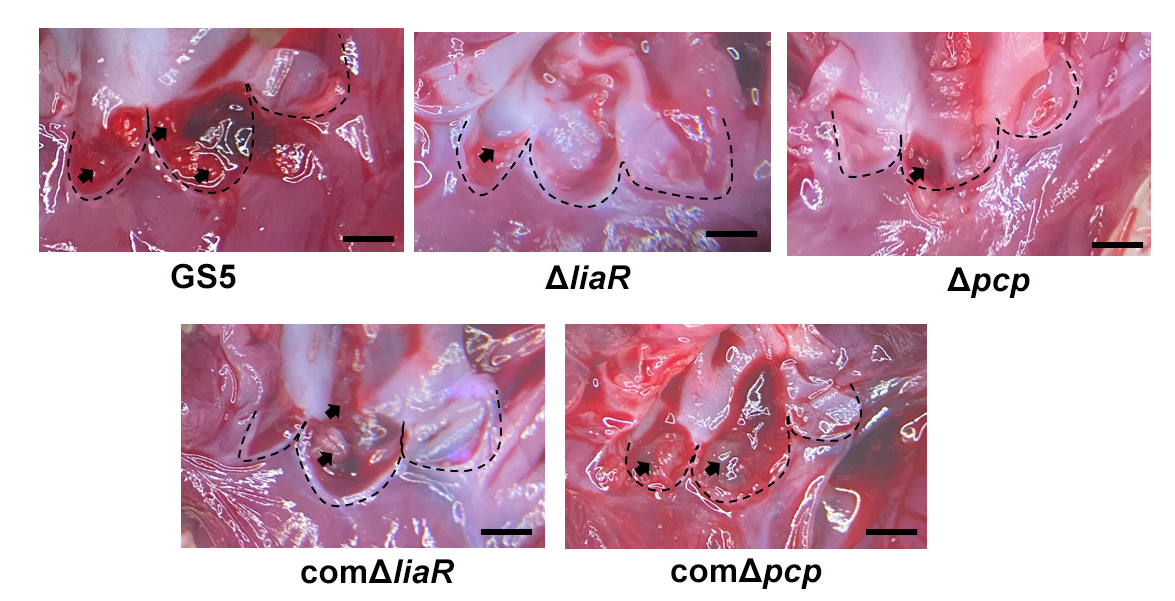

Supplement: S1 Fig — S. mutans GS5 wild-type, ΔliaR, Δpcp, comΔliaR, or comΔpcp strains were intravenously injected into experimental IE rat models. Photographs of the vegetation formation on the heat valve are shown. The markers represent the valves (dash lines) and vegetations (black arrows). Scale bars represent 1 mm. (TIF) [file ppat.1009289.s002.tif]

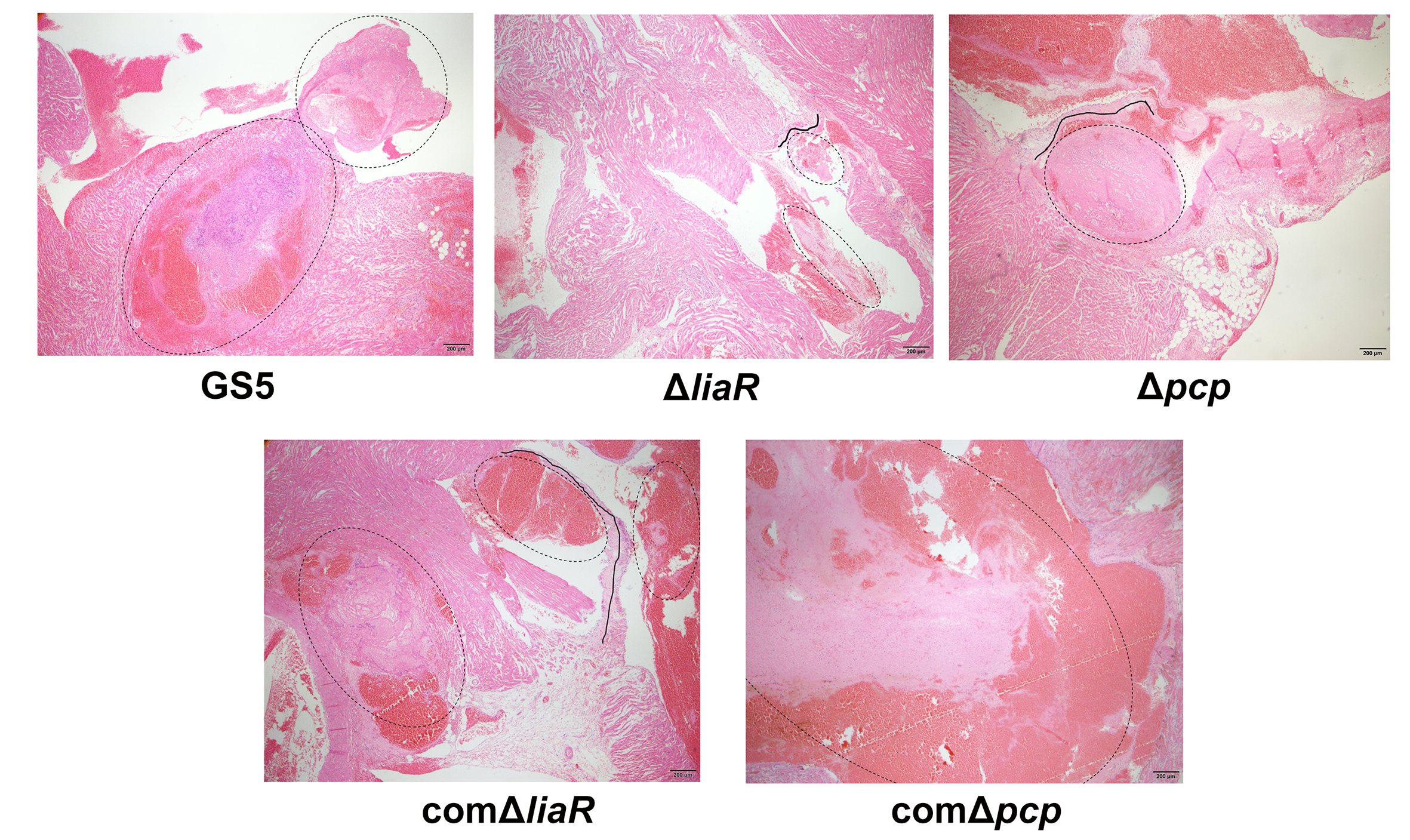

Supplement: S2 Fig — S. mutans GS5 wild-type, ΔliaR, Δpcp, comΔliaR, or comΔpcp strains were intravenously injected into experimental IE rat models and the rats were sacrificed 24 h post-infection. Heart tissue was then collected for histopathology staining. The markers represent the valves (solid lines) and the vegetations (dash circles). Scale bars represent 200 μm. (TIF) [file ppat.1009289.s003.tif]

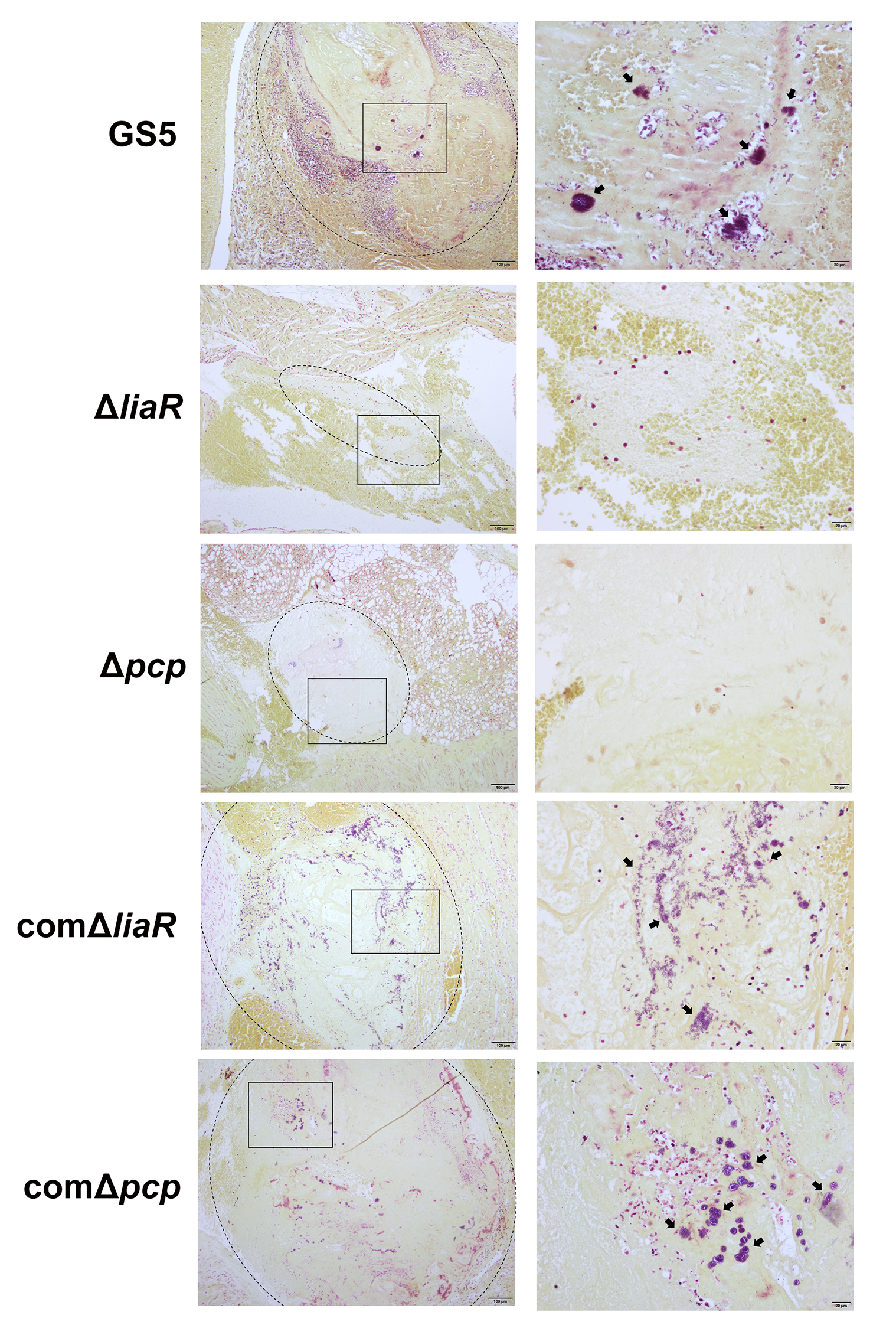

Supplement: S3 Fig — S. mutans GS5 wild-type, ΔliaR, Δpcp, comΔliaR, or comΔpcp strains were intravenously injected into experimental IE rat models and the rats were sacrificed 24 h post-infection. Vegetation was subsequently collected for gram staining. The markers represent the vegetations (dash circle) and the bacteria (black arrows). The bacteria cannot be easily detected by gram staining in the ΔliaR and Δpcp samples. Scale bars represent 100 μm (left panels) and 20 μm (right panels). (TIF) [file ppat.1009289.s004.tif]

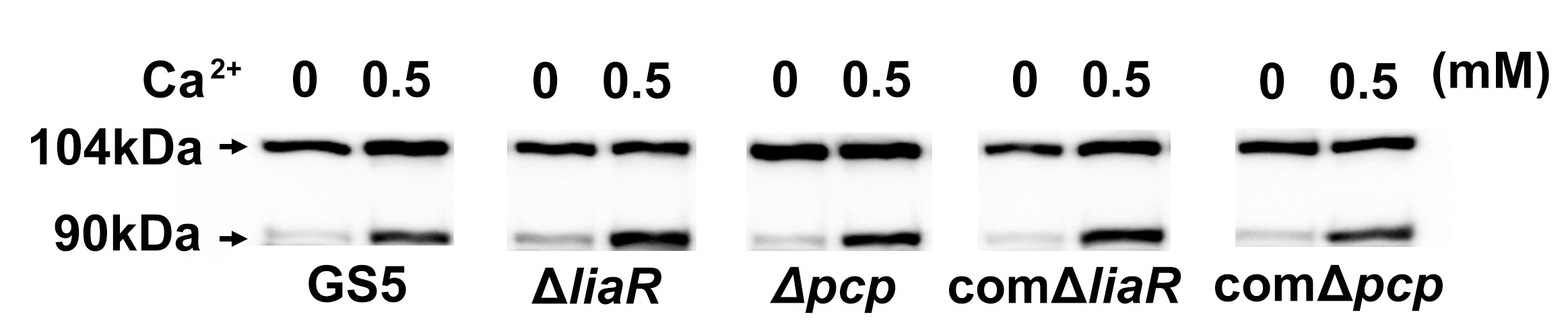

Supplement: S4 Fig — S. mutans GS5 wild type, ΔliaR, Δpcp, comΔliaR and comΔpcp were cultured in BHI medium in the absence (lane 1) or presence (lanes 2) of the indicated concentrations of CaCl2, and the bacterial surface proteins were extracted by 4% SDS. AtlA expression and the mature form of AtlA (90 kDa) were detected by Western blot analysis. (TIF) [file ppat.1009289.s005.tif]

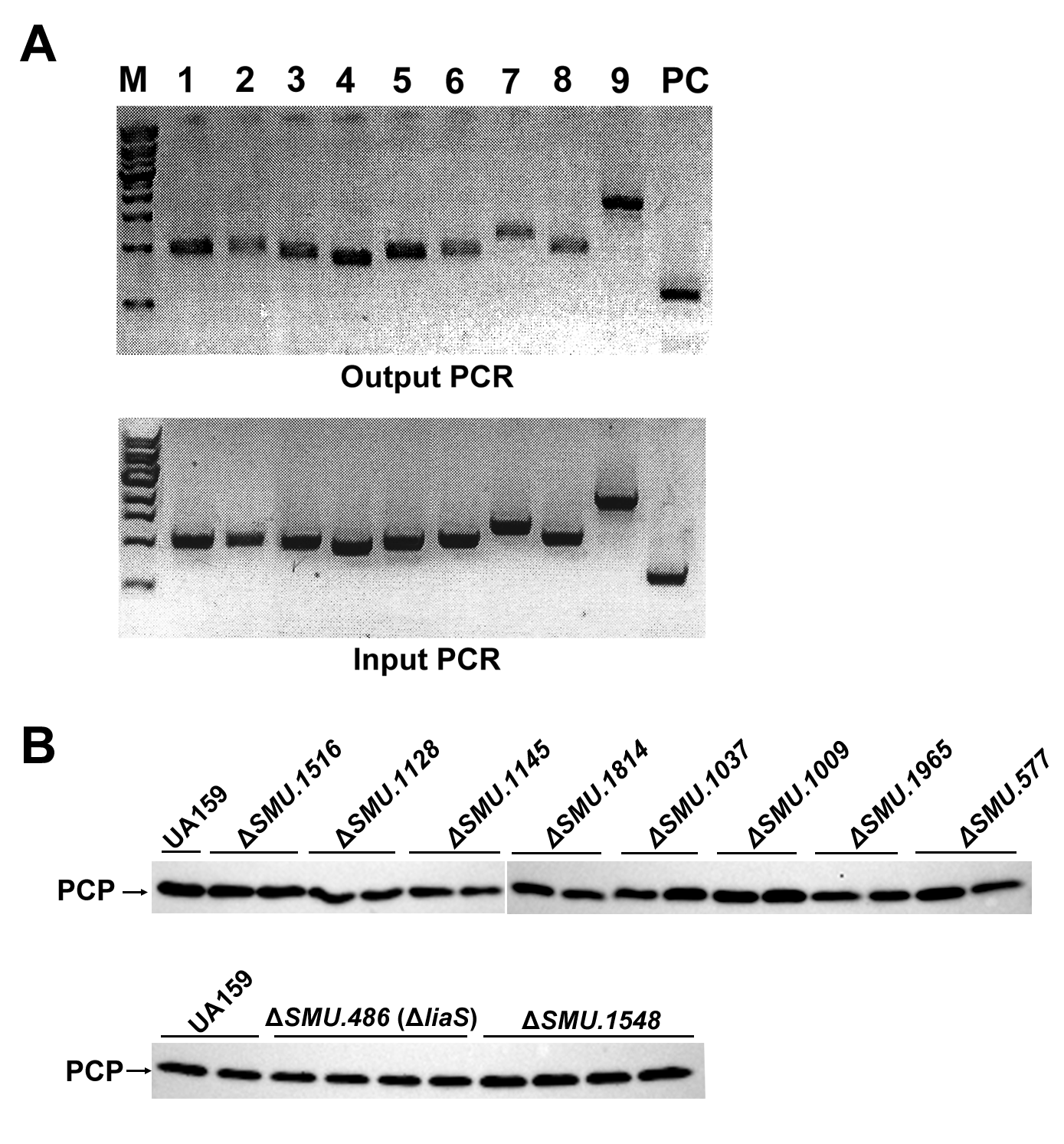

Supplement: S5 Fig — (A) Identification of the sensor kinase involved in bacterial biofilm formation in in vivo survival assay. The upper panel is a representative PCR result of injected bacterial mixtures (input PCR); the bottom panel is a representative PCR result of bacteria colonized inside the vegetation. M, DNA marker; lane 1, the PCR result using the primers for detecting SMU.1128-deficient mutant; lane 2, SMU.1145; lane 3, SMU.1814; lane 4, SMU.1037; lane 5, SMU.1009; lane 6, SMU.1965; lane 7, SMU.577; lane 8, SMU.486 (liaS); lane 9, SMU.1548; and PC, positive control, PCR product of 16S rRNA gene. (B) Surface protein of S. mutans UA159 wild type and the isogenic mutant strains of sensor kinases were extracted by 4% SDS, and the expression of PCP were detected by Western blot analysis. (TIF) [file ppat.1009289.s006.tif]

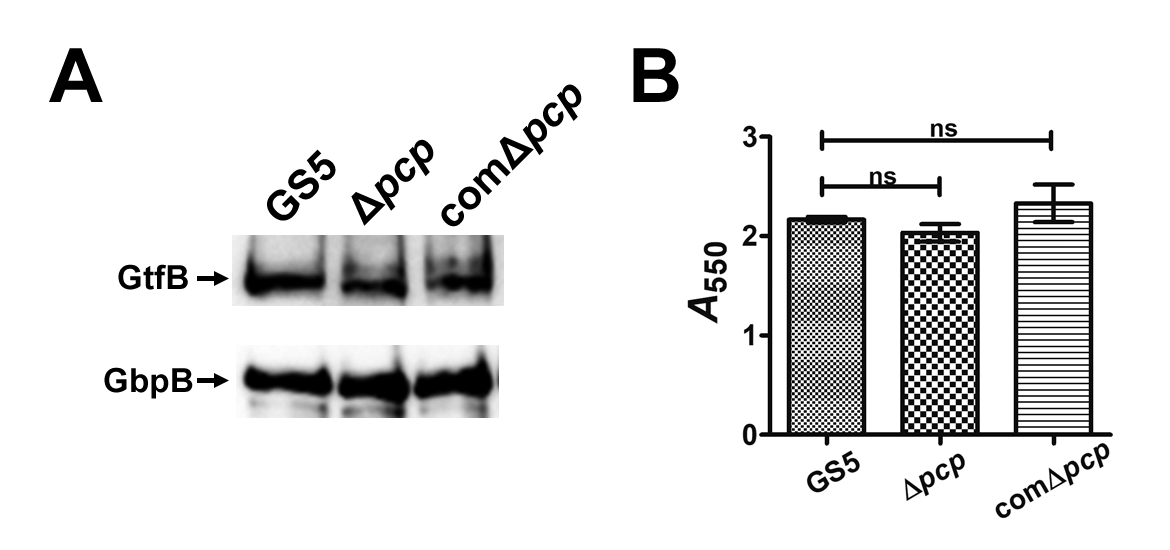

Supplement: S6 Fig — (A) Surface protein of S. mutans GS5 wild type, Δpcp and comΔpcp were extracted by 4% SDS, and the expression of glucosyltransferase-I (GtfB) and glucan binding protein B (GbpB) were detected by Western blot analysis. (B) Biofilms of S. mutans GS5 wild type, Δpcp, and comΔpcp cultured in BHI medium containing 1% sucrose were stained with 0.1% crystal violet and the absorbance quantified at 550 nm. Data are expressed as the means ± standard deviations of triplicate experiments; ns, not significant by 1-way analysis of variance. (TIF) [file ppat.1009289.s007.tif]
